# Enhanced Bioactive Peptide Release from Pre-Hydrolysed Pea Protein: Impact of Pepsin Digestion on Antidiabetic and Antihypertensive Functions

**DOI:** 10.3390/foods14193306

**Published:** 2025-09-24

**Authors:** Arig Elbira, Alan Javier Hernández-Álvarez, Christine Boesch

**Affiliations:** 1 School of Food Science and Nutrition, Faculty of Environment, University of Leeds, Leeds LS2 9JT, UK; fs19aaae@leeds.ac.uk (A.E.); a.j.hernandezalvarez@leeds.ac.uk (A.J.H.-Á.); 2 National Alternative Protein Innovation Centre (NAPIC), UK

**Keywords:** pea protein, hydrolysates, protein hydrolysis, α-amylase, α-glucosidase, dipeptidyl peptidase IV (DPP-IV), angiotensin-converting enzyme (ACE), plant-based proteins, ultrafiltration

## Abstract

There is increasing interest in the health-promoting potential of plant protein-derived peptides for managing metabolic disorders. This study investigated the impact of pepsin digestion on pre-hydrolysed versus non-hydrolysed pea protein. Pepsin digestion resulted in a higher degree of hydrolysis in pre-hydrolysed samples (64%) compared to the non-hydrolysed samples (~40%). The pepsin hydrolysates from the pre-hydrolysed protein showed stronger inhibition of key metabolic enzymes compared to non-hydrolysed samples. After ultrafiltration to enrich peptides <10 kDa, inhibition of α-amylase, α-glucosidase, and ACE was markedly enhanced, achieving a maximum of 44.5%, 54% and 95%, respectively. Peptidomic analysis identified unique peptide sequences in the ultrafiltered pre-hydrolysed fraction, in silico prediction confirmed their bioactive potential. These findings demonstrate enhanced bioactivity in pre-hydrolysed pea protein samples following pepsin hydrolysis, which was most evident in the ultrafiltrated fractions. Overall, this approach highlights the relevance of enzymatic hydrolysis and peptide enrichment strategies in developing functional ingredients to support glucose regulation and cardiovascular health.

## 1. Introduction

A growing body of research is focused on valorising plant-derived proteins for their potential health benefits, with particular attention to the generation of bioactive peptides. These peptides, typically ranging from 2 to 20 amino acids in length, are released from parent proteins during enzymatic hydrolysis and have shown promise as functional ingredients to manage chronic diseases such as type 2 diabetes, hypertension, and cardiovascular disease [1,2]. Among various plant sources, oilseed meals and legumes have been widely explored, yet more recently, pea protein has emerged as a sustainable and nutritionally valuable protein source with demonstrated bioactivity potential.

Enzymatic hydrolysis is the preferred method for generating bioactive peptides due to its mild conditions and enzyme specificity, which minimises the risk of reducing protein quality [3,4]. Hydrolysis can release a heterogeneous mixture of peptides with varying molecular weights and sequences, depending on the specificity of the protease used and the amino acid sequence [5]. These peptides can exert biological effects such as antioxidant, antihypertensive, antimicrobial, anti-inflammatory, antidiabetic, among others, offering functional opportunities in the development of health-promoting foods [6,7].

Yellow pea (*Pisum sativum* L.), in particular, has gained increasing interest not only due to its high protein content and amino acid profile but also its low allergenicity and environmental sustainability. Hydrolysed pea protein has been reported to yield bioactive peptides that can inhibit carbohydrate digesting enzymes such as α-amylase and α-glucosidase, key enzymes involved in starch digestion as well as glucose absorption [8,9]. Inhibition of these enzymes can slow the breakdown of carbohydrates, contributing to reduced postprandial blood glucose spikes—a mechanism especially relevant for the dietary prevention and management of type 2 diabetes [10].

The extent of hydrolysis and the functional properties of hydrolysates can vary based on protein structure, protease specificity, and hydrolysis conditions such as temperature, pH, enzyme/substrate ratio and duration [11,12]. For example, pepsin, a gastric protease, is commonly used to simulate human gastric digestion and has been shown to generate bioactive peptides from various plant proteins [13]. However, variations in the level of protein pre-processing—such as partial hydrolysis or targeted structural modifications—can significantly influence the efficiency of enzymatic hydrolysis and the profile of generated peptides. Furthermore, while whole hydrolysates are a heterogeneous mixture of different sizes of peptides and amino acids, low molecular weight fractions (<10 kDa) have often been associated with greater bioactivity, particularly for ACE inhibition, due to their shorter chain length and greater conformational flexibility that facilitates strong interactions with enzyme active sites [14,15]. Additionally, the high abundance of small peptides in these fractions may exert synergistic effects, further amplifying their inhibitory potential.

In addition to molecular weight, the specific amino acid sequence and hydrophobicity are critical factors that influence peptide bioactivity, predominantly in relation to enzyme inhibition [16,17]. Specific amino acid motifs—particularly residues such as Pro, Tyr, Phe, and Leu at the N- or C-terminal—have been shown to enhance binding affinity to enzymes like ACE and DPP-IV [18,19]. While in silico prediction tools can provide useful insights into the potential bioactive sequences released by enzymatic hydrolysis, experimental validation remains essential. Peptidomic techniques, such as LCMS/MS coupled with bioinformatic analysis, enable the identification and characterisation of peptide sequences present in a hydrolysate mixture [20,21]. These analytical approaches are crucial for confirming the presence of bioactive peptides and elucidating the mechanisms underlying observed in vitro enzyme inhibitory effects in protein hydrolysates [22].

The current study was designed to provide a comprehensive understanding of how pepsin-mediated hydrolysis impacts both native and pre-hydrolysed pea proteins in terms of their ability to generate peptides with antidiabetic and antihypertensive potential.

## 2. Materials and Methods

### 2.1. Materials and Chemicals

Pea protein samples were obtained from two sources: NHP1-R was purchased online from Pulsin (Gloucestershire, UK), while NHP2-R (NUTRALYS^®^ S85F) and PHP-R (NUTRALYS^®^ S85 Plus N) were kindly provided by ROQUETTE (NUTRALYS^®^, Lestrem, France). NHP1-R and NHP2-R represent untreated pea protein isolates, which allow for baseline comparisons, whereas PHP-R is a commercially pre-hydrolysed pea protein sample. Pierce^TM^ BCA Protein Assay Kit, Pierce^TM^ Protein Concentrators PES (10 K MWCO), and Pierce^TM^ Quantitative Fluorometric Peptide Assay Kit were obtained from Thermo Fisher Scientific (Waltham, MA, USA). Criterion^TM^ TGX Precast Gels, Pre-Stained Protein Molecular Weight Marker, SDS Laemmli Sample Buffer, and 4× SDS Running Buffer were purchased from Bio-Rad Laboratories (Hercules, CA, USA). Pepsin from porcine gastric mucosa, 2,4,6-trinitrobenzenesulfonic acid solution (TNBS), α-glucosidase from Saccharomyces cerevisiae, p-nitrophenyl-α-D-glucopyranoside, Acarbose, α-amylase from Aspergillus oryzae, Gly-Pro-p-nitroanilide (Gly-Pro-pNA), Dipeptidyl Peptidase-IV (DPP-IV) enzyme, Diprotin A (Ile-Pro-Ile), Angiotensin-Converting Enzyme (ACE), Captopril, and N-[3-(2-Furyl) acryloyl]-Phe-Gly-Gly (FAPGG) were purchased from Sigma-Aldrich (St. Louis, MO, USA).

### 2.2. Characterisation of Pea Protein Products

The protein content of three pea protein samples—NHP1-R and NHP2-R (2 types of non-hydrolysed pea protein) and PHP-R (pre-hydrolysed)—was determined using Dumas combustion method, which measures total nitrogen [23]. EDTA was used as a high-nitrogen standard, and rice flour as a low-nitrogen standard.

To analyse the molecular weight distribution, SDS-PAGE (Sodium Dodecyl Sulfate–Polyacrylamide Gel Electrophoresis) was performed using a 16% Tricine-SDS-PAGE gel. Samples were prepared in 2× Laemmli buffer, briefly centrifuged at 13,000× *g* for 1 min, and 10 µL of each sample was loaded per well, corresponding to 15 µg total protein. Electrophoresis was conducted at 200 V for 1 h. The analysis was performed under both reducing and non-reducing conditions. Following separation, gels were stained with Coomassie Brilliant Blue, destained using standard procedures, and preserved for analysis. A wide-range molecular weight marker was included to estimate the molecular weight of the protein bands [24,25].

Water solubility of the protein samples was evaluated across a pH range of 2 to 12 using a modified method based on Betschart et al. [26]. Protein dispersions (200 mg in 20 mL distilled water) were adjusted to the desired pH using 1 N HCl or 1 N NaOH. The samples were stirred continuously for 30 min, then centrifuged at 3500× *g* for another 30 min. The supernatant was collected, and soluble protein content was quantified using the BCA assay [27]. Briefly, 10 µL of the sample was mixed with 200 µL of BCA reagent, incubated at 37 °C for 30 min, and absorbance was read at 560 nm using BSA as the standard. Solubility was expressed as the percentage of soluble protein relative to the total protein content of the original sample.

### 2.3. Preparation of Crude Pea Protein Hydrolysates and <10 kDa Fractions

Each protein sample was suspended in a distilled water solution at a final protein concentration of 50 mg/mL, and the pH was adjusted to 2. Pepsin (specific activity ≥ 2500 U/mg protein, measured using haemoglobin as substrate at pH 2.0 and 37 °C) was added at enzyme-to-substrate (E/S) ratios of 1:50 and 1:100 (*w*/*w*), and the mixture was incubated for 2 h at 37 °C. Samples were collected at different time points (0, 5, 15, 30, 45, 60, 120 min) during the enzymatic hydrolysis to assess further. All protein hydrolysates were deactivated by immersing them in boiling water for 10 min and centrifuged at 13,000× *g* for 15 min at 4 °C to remove non-digested protein. After that, the pH of the resulting supernatant was subsequently adjusted to 7.0. Low molecular weight fractions (<10 kDa) were obtained using ultrafiltration with 10 kDa molecular weight cut-off membranes (10 K MWCO). The crude hydrolysates of non-hydrolysed protein samples and the pre-hydrolysed protein were designated as NHP1-CH, NHP2-CH, and PHP-CH, respectively, while their corresponding <10 kDa fractions were referred to as NHP1-F, NHP2-F, and PHP-F. All supernatants were lyophilized and stored at −20 °C for subsequent analyses.

#### 2.3.1. Assessment of Degree of Hydrolysis (DH)

The DH was assessed using the TNBS method with L-leucine as a reference standard, following the protocol outlined by Ma et al., [28]. 100 µL of each sample was then treated with 50 µL 0.1% TNBS solution to react with the released amino groups. After incubation at 37 °C for 2 h, the reactions were stopped by adding 50 µL 10% SDS and 50 µL 1 M HCl. The absorbance of the resulting solution was measured at 340 nm. To determine the total number of peptide bonds present in the sample (*h_tot_*), a completely hydrolysed sample was generated in 6 M HCl using a microwave at 100 °C for 45 min. The DH percentage was then calculated using the following formula:

(1) $$\;DH=\;h/h_{tot}\times 100\%$$

where *h* = number of hydrolysed peptide bonds in a sample and *h_tot_* = the total number of equivalent protein peptide bonds.

#### 2.3.2. Quantification of Released Peptides

Peptide concentration was determined using an amine-reactive fluorometric assay [29]. Briefly, 10 μL of each sample was mixed with 70 μL assay buffer and 20 μL reagent in a fluorescence-compatible plate, incubated for 5 min at room temperature, and measured at Ex 390 nm/Em 475 nm. A standard peptide calibration curve was used for quantification [30].

#### 2.3.3. Determination of Soluble Protein

The soluble protein content of each hydrolysate at different hydrolysis times was measured using the BCA assay, as mentioned above [27]. The amount of soluble protein present in each sample was expressed as mg/mL.

### 2.4. Enzyme Inhibitory Properties

#### 2.4.1. Measurement of α-Amylase Inhibitory Activity

α-Amylase inhibitory activity was evaluated following the method of Zulfiqar et al., with slight modifications [31]. Pea protein hydrolysates and membrane fractions were dissolved in 50 mM sodium phosphate buffer (pH 6.9) at various concentrations (2–20 mg/mL). Then, 50 μL of each sample was mixed with 100 μL of α-amylase solution and incubated at 37 °C for 10 min. Afterwards, 50 μL of 2 mM CNP-G3 (in the same buffer) was added, and the mixture was incubated for another 10 min at 37 °C. Absorbance was measured at 405 nm. Acarbose was used as a positive control, and results were expressed as a percentage of the uninhibited control.

#### 2.4.2. Measurement of α-Glucosidase Inhibitory Activity

The inhibitory properties of pea protein hydrolysates and their fractions on α-glucosidase activity were assessed through a microplate-based assay, following the methodology of Zulfiqar et al. [32]. In brief, 100 μL of a protein hydrolysate solution at a concentration of (2–20 mg/mL) was mixed with 50 μL of 0.5 U/mL α-glucosidase solution (dissolved in 0.1 M PBS, pH 7.0), and the mixture was pre-incubated at 37 °C for 10 min. Subsequently, 50 μL of a 2.5 mM pNPG substrate was added to initiate the reaction, and the absorbance at 405 nm was recorded over a 10 min period. Acarbose served as a positive control. The results are expressed as a percentage relative to the non-inhibited control.

#### 2.4.3. Measurement of DPP-IV Inhibitory Activity

DPP-IV inhibitory activity was assessed using a colorimetric assay according to a previously described method by Han at al. [33] with minor modifications. Pea protein hydrolysates and fractions were diluted in 0.1 M Tris buffer (pH 8.0) and 25 μL was added to each well of transparent 96-well plate (1–5 mg/mL), along with negative controls (buffer). Substrate solution (25 μL, 10 mM) was added, and the plate incubated at 37 °C for 10 min. The reaction was initiated by adding 50 μL of DPP-IV enzyme solution (500 U/mL), and absorbance was measured at 405 nm over 60 min in 5 min intervals. Diprotin A was used as a positive control. The results are expressed as a percentage compared to non-inhibited control.

#### 2.4.4. Measurement of ACE Inhibitory Activity

ACE inhibitory activity was measured using a kinetic colorimetric assay with FAPGG as the substrate [34]. To ensure accurate detection of inhibition while avoiding interference from higher sample concentrations, pea protein hydrolysates and their fractions were prepared at 1–5 mg/mL. In addition, Preliminary tests showed that increasing the concentration beyond this range did not result in any further increase in enzyme inhibition. Samples were dissolved in 50 mM Tris-HCl Buffer (pH 8.3) containing 0.3 M NaCl. For each reaction, 20 μL of the sample solution was mixed with 20 μL of ACE solution (100 mU/mL). The reaction was initiated by adding 100 μL of 1 mM FAPGG, and absorbance at 340 nm was recorded in 1 min intervals for 30 min at 37 °C. ACE inhibition was calculated based on the change in absorbance (ΔA) relative to the negative control, and results were expressed as percentage inhibition. Captopril was used as a positive control in the assay.

### 2.5. Peptidomic Profiling of Pea Protein Hydrolysates/Fractions

Four pea protein samples (NHP1-F, NHP2-F, PHP-CH, and PHP-F) were selected based on their highest in vitro enzyme inhibitory activity to identify the peptide sequences that could be responsible for enzyme inhibition. All samples were collected at 120 min of enzymatic hydrolysis with an E/S ratio of 1:50 to ensure consistent comparison. These conditions resulted in the highest degree of hydrolysis (DH) and peptide release across all samples.

Prior to analysis, the samples were desalted using C18 solid-phase extraction (SPE) to remove salts and other interfering substances. Peptides were analysed by liquid chromatography–tandem mass spectrometry (LC-MS/MS) at the University of York using an EvoSep One UHPLC system coupled to a Bruker timsTOF HT mass spectrometer. Data acquisition was performed in parallel accumulation–serial fragmentation data-dependent acquisition mode (PASEF-DDA) using a 60 samples-per-day (SPD) gradient elution profile optimised for peptide separation.

MS/MS data were searched using FragPipe against the *Pisum sativum* (pea) protein subset of the UniProt database, supplemented with common proteomic contaminants. Peptide identification was based on pepsin-like enzymatic cleavage specificity, defined as cleavage C-terminal to phenylalanine (F) or leucine (L), with allowance for semi-specificity—requiring cleavage specificity at only one terminus of the peptide. Peptide-spectrum matches were filtered to a 1% false discovery rate (FDR), assessed against a decoy database. Proteins were retained only if identified by at least two unique peptides.

Raw mass spectrometry data, peak lists and peptidomic results are deposited in MassIVE (MSV000098336) [doi:10.25345/C5959CM40] and referenced in ProteomeXchange (PXD065499). Data are available from the following ftp location: ftp://MSV000098336@massive-ftp.ucsd.edu (accessed on 6 August 2025).

### 2.6. In Silico Bioactivity Prediction of Peptides Identified from Pea Protein Hydrolysate/Fractions

Following peptide identification, the ten most frequently occurring peptides across all samples were selected for in silico prediction of potential bioactivity. PeptideRanker (http://distilldeep.ucd.ie/PeptideRanker/ (accessed on 6 August 2025)) was used to assign a probability score to each peptide based on its likelihood of exhibiting bioactivity, with a threshold score of 0.5 considered indicative of potential functionality. To further investigate specific enzyme inhibitory properties, peptide sequences were analysed using the BIOPEP-UWM database (http://www.uwm.edu.pl/biochemia/index.php/en/biopep (accessed on 6 August 2025)), which enables comparison of input peptides against known bioactive sequences. The database was used to predict potential inhibitory activity against glycaemic-regulating enzymes, including α-amylase, α-glucosidase, and DPP-IV, as well as ACE [35]. In addition, key structural features of these peptides, such as sequence length, molecular weight, hydrophobicity, hydrophobic residue content, and net charge at pH 7, were analysed to explore potential relationships between peptide structure and predicted biological activity.

### 2.7. Statistical Analysis

All assays were conducted in triplicate and data analysed as the mean values ± standard deviation. The mean values were examined using analysis of variance (ANOVA) and then compared using Duncan’s multiple range test with significant differences accepted at *p* < 0.05. All analyses were conducted using GraphPad Prism version 10.

## 3. Results and Discussion

### 3.1. Characterisation of Raw Pea Protein Samples

Variations in pre-hydrolysis protein profiles among plant proteins can significantly influence their enzymatic digestibility and the generation of bioactive peptides [4]. In this study, three pea protein samples—NHP1-R, NHP2-R, and PHP-R—presenting with different hydrolysis profiles were evaluated for their protein solubility and molecular weight distributions, revealing distinct structural characteristics that may affect their bioactive potential.

**Figure 1:**
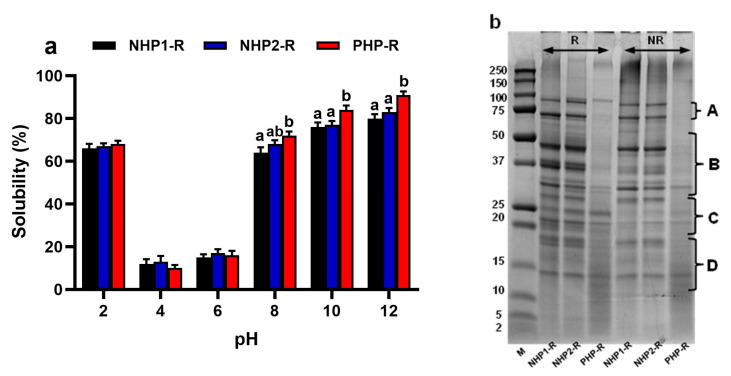
(**a**) Solubility profiles of non-hydrolysed pea proteins (NHP1-R, NHP2-R) and pre-hydrolysed pea protein (PHP-R) measured across a pH range (2–12). Data are expressed as mean ± SD (*n* = 3). Different letters indicate statistically significant differences between samples at each pH level (*p* < 0.05, one-way ANOVA with Tukey’s test), (**b**) SDS-PAGE analysis of NHP1-R, NHP2-R, and PHP-R samples under reducing (R) and non-reducing (NR) conditions. Each sample was loaded at 15 µg protein. M = molecular weight marker (kDa). A: Convicilin (77.9 kDa, 72.4 kDa); B: Vicilin (35–50 kDa) and Legumin acidic subunit (~41 kDa); C: Legumin basic subunits (~23 kDa); D: Albumin (10–16 kDa).

While all three proteins had comparable protein contents (ranging from 77% to 79.1%), their solubility diverged considerably across the pH spectrum. As shown in Figure 1a, solubility increased at both acidic and alkaline pH levels for all samples, consistent with the general behaviour of globular plant proteins due to electrostatic repulsion away from the isoelectric point [36,37]. However, PHP-R showed a significantly higher solubility at pH 12 (91%) compared to NHP1-R (78%) and NHP2-R (81%) (*p* < 0.01). This enhanced solubility can be attributed to partial enzymatic hydrolysis, which disrupts the compact tertiary structure of globular proteins, leading to unfolding and exposure of hydrophilic residues on the protein surface [38,39]. Additionally, hydrolysis reduces the molecular weight and hydrophobicity of protein aggregates, resulting in decreased intermolecular interactions and less tendency to aggregate, both of which promote solubility [40,41]. Similar solubility trends have been observed in other legume proteins, such as soy and lentil isolates, which show minimal solubility near their isoelectric points and improved solubility under alkaline conditions [38]. Notably, the solubility of PHP-R at high pH exceeds typical values reported for untreated pea protein isolates (<80% at pH 12), suggesting that pre-hydrolysis significantly enhances solubility beyond the native structural limits of pea protein [22,42].

Figure 1b shows the SDS-PAGE profiles of the three proteins tested—NHP1-R, NHP2-R, and PHP-R, under both reducing and non-reducing conditions. The native proteins (NHP1-R and NHP2-R) exhibited prominent bands within the 25–75 kDa range, consistent with typical polypeptide subunits of pea storage proteins, particularly vicilin, legumin, and convicilin. As previously described by Shevkani et al., [43], bands around 70–75 kDa are attributable to convicilin (Band A). While bands near 43–53 kDa correspond to vicilin, a trimeric protein made up of α-, β-, and γ-subunits. The acidic (~37 kDa) and basic (~22–25 kDa) subunits of legumin, a hexameric storage protein, were also observed (Bands D and E, respectively). A band around 32 kDa was present in NHP1-R and NHP2-R, which may correspond to phytohemagglutinin (lectin), as also reported by Shevkani et al. and others [44,45].

Under reducing conditions, the band resolution improved, particularly in the NHP samples, indicating the dissociation of disulfide-linked aggregates and better separation of individual subunits. This also supports the presence of covalent (disulfide) bonding within legume storage proteins [44]. In contrast, PHP-R showed fewer defined bands and a smear of low molecular weight fragments (<25 kDa), indicating partial hydrolysis, consistent with earlier findings on enzymatically treated legume proteins [42,46]. While vicilin bands were mostly absent, a faint convicilin band (~72 kDa) remained, likely due to its higher resistance to proteolysis or lower accessibility of cleavage sites [43]. The increased intensity of bands at 10–16 kDa, could be attributed to the presence of albumins or protease inhibitors; additionally, the accumulation of smaller peptides post-hydrolysis could be observed. [45].

### 3.2. PHP-CH Exhibits Enhanced Pepsin Digestibility, Peptide Release and Solubility

After pepsin hydrolysis, all three protein samples were evaluated as crude hydrolysates, referred to as NHP1-CH, NHP2-CH, and PHP-CH. Among them, PHP-CH consistently exhibited superior pepsin accessibility, peptide yield, and solubility. These improvements are likely due to pre-partial hydrolysis, which may have unfolded the protein structure and increased the exposure of pepsin cleavage sites, facilitating more efficient digestion [11].

**Figure 2:**
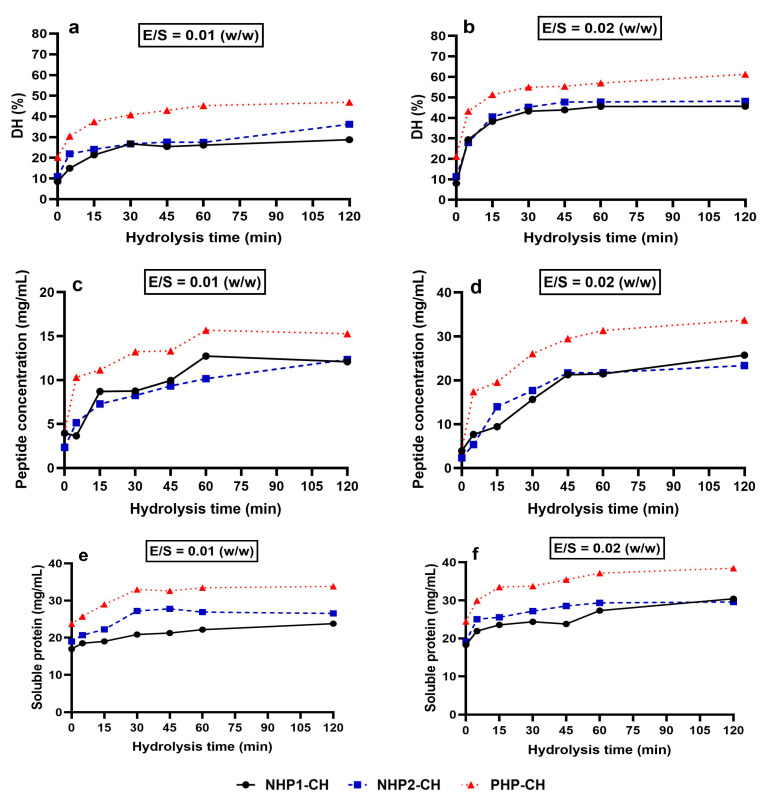
Degree of hydrolysis (DH%) (**a**,**b**), peptide concentration (µg/mL) (**c**,**d**), and soluble protein content (%) (**e**,**f**) of non-hydrolysed protein samples (NHP1-R, NHP2-R) and pre-hydrolysed protein sample (PHP-R) over a 120 min pepsin hydrolysis period. (**a**,**c**,**e**) correspond to enzyme-to-substrate (E/S) ratio 1:50 (*w*/*w*), while (**b**,**d**,**f**) correspond to E/S ratio 1:100 (*w*/*w)*. Time point 0 represents the raw protein samples (NHP1-R, NHP2-R, and PHP-R). After digestion start (t > 0), the resulting digesta are referred to as crude hydrolysates (NHP1-CH, NHP2-CH, and PHP-CH). Data are presented as mean ± standard deviation (*n* = 3).

The degree of hydrolysis (DH) increased over time for all samples under both enzyme-to-substrate (E:S) ratios tested (1:50 and 1:100), with PHP-CH consistently showing the highest DH at each time point. This trend is visually evident in Figure 2a,b, where the PHP-CH sample (triangle markers) displays a steeper initial rise and a higher final DH under both enzyme concentrations. At 120 min, PHP-CH reached 61% DH at E:S 1:50, compared to 48% for NHP1-CH and 45% for NHP2-CH. A similar pattern was observed at E:S 1:100, confirming that a higher E:S ratio promotes more extensive hydrolysis, although PHP-CH maintained its advantage regardless of enzyme concentration. Notably, PHP-CH also showed a DH of 20% at time zero, indicating that enzymatic cleavage had already occurred during prior processing. These findings align with results reported by He et al. [47], who showed that pre-hydrolysis of plant proteins can enhance their susceptibility to subsequent enzymatic digestion.

The release of peptides followed a comparable trend. At 120 min and E:S 1:50, PHP-CH released 48,000 µg/mL of peptides, significantly exceeding that of NHP1-CH (34,000 µg/mL) and NHP2-CH (31,000 µg/mL) (Figure 2c). A similar pattern was observed at E:S 1:100 (Figure 2d), although total peptide yield was reduced across all samples. This confirms that a lower E:S ratio leads to less efficient cleavage but does not alter the relative differences between the samples. These results are consistent with previous findings that pre-hydrolysis enhances enzymatic peptide liberation by disrupting compact protein structures [48,49].

After hydrolysis, PHP hydrolysates maintained a significantly higher solubility (91%) than those from NHP1 and NHP2 (71% and 72%, respectively) (Figure 2e,f). This aligns with findings by previous researchers who reported that limited hydrolysis of legume proteins increases surface hydrophilicity and solubility, thereby enhancing their functionality in food applications [18,50].

Collectively, these findings suggest that pre-hydrolysis enhances the susceptibility of pea protein to enzymatic digestion, leading to a more efficient release of bioactive peptides and improved solubility. Such characteristics are essential for developing functional food ingredients with potential health benefits, particularly for targeting digestive and metabolic enzyme inhibition. These results are consistent with prior studies indicating that controlled hydrolysis can improve both the nutritional and therapeutic potential of plant protein ingredients [51].

### 3.3. Inhibition of Carbohydrate-Digesting Enzymes by Pea Protein Hydrolysates and Peptide Fractions

**Figure 3:**
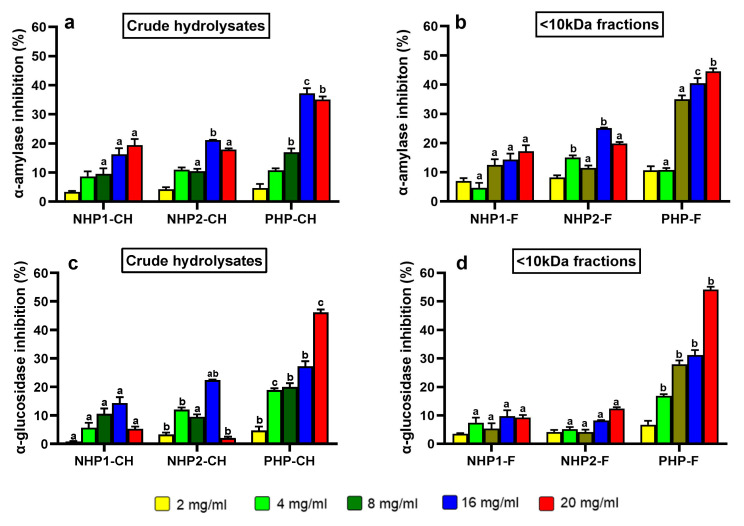
Inhibitory activity of crude hydrolysates NHP1-CH, NHP2-CH, and PHP-CH and <10 kDa peptide fractions of NHP1-F, NHP2-F, and PHP-F against α-amylase (**a**,**b**) and α-glucosidase (**c**,**d**). Each bar represents the mean ± standard deviation (SD), (*n* = 3). Samples were tested at five concentrations. Different letters indicate significant differences (*p* < 0.05) between protein samples at the same concentration.

One of the most compelling findings of this study was the ability of crude hydrolysates and peptide fractions of pre-hydrolysed pea protein to inhibit key carbohydrate-digesting enzymes, particularly α-amylase and α-glucosidase. The α-amylase inhibitory activity of both crude hydrolysates and their ultrafiltered fractions showed a clear dose-dependent pattern, as shown in Figure 3a. Across all concentrations tested, PHP-CH consistently demonstrated significantly higher α-amylase inhibition compared to NHP1-CH and NHP2-CH, with the highest inhibition observed at 16 mg/mL (approximately 37%), while NHP1-CH and NHP2-CH showed only 19.4% and 21.1% inhibition, respectively.

This trend was even more pronounced in the ultrafiltered peptide fractions (Figure 3b). At 20 mg/mL, PHP-F exhibited 44.5% inhibition, significantly higher than NHP2-F (25%) and NHP1-F (17.4%). These results indicate that pre-hydrolysis enhances the release of peptides with greater α-amylase inhibitory capacity [13,17].

Although acarbose achieved much higher inhibition (~70–85%) at very low concentration, this is expected given its purity and pharmacological potency. In contrast, protein hydrolysates are complex peptide mixtures, where structural diversity and peptide–peptide or peptide–enzyme interactions can influence bioactivity in non-linear ways [39].

In the case of α-glucosidase, a dose-dependent response was again observed, but PHP stood out even more clearly. At the highest tested concentration (20 mg/mL), PHP-CH achieved 46% inhibition, while NHP1-CH and NHP2-CH showed minimal activity—only 14% and 22% inhibition at 16 mg/mL (Figure 3c). Interestingly, the ultrafiltered <10 kDa fraction of PHP showed an enhanced activity, reaching 54% inhibition. These results align with previous findings on soybean and chickpea peptides, where <10 kDa fractions showed stronger α-glucosidase inhibition, typically 40–60% at 10–20 mg/mL (Figure 3d), highlighting the enhanced bioactivity of low molecular weight peptides [9,52,53]. On the other hand, NHP1-F and NHP2-F showed reduced inhibitory potential, at only 9% and 12%, respectively. The reduced α-glucosidase inhibition in NHP1-F and NHP2-F suggests that the ultrafiltration step could possibly separate the medium-sized peptides responsible for the observed activity in the crude hydrolysates. Therefore, the improved performance of PHP-F in this study may be attributed not only to a higher abundance of small peptides, but also to the presence of specific bioactive sequences generated through pre-hydrolysis and efficient pepsin digestion [52].

Taken together, these findings indicate that pre-hydrolysed pea protein is a promising source of bioactive peptides capable of modulating carbohydrate metabolism. The strong inhibition observed in the <10 kDa PHP fraction, especially against α-glucosidase, supports the concept that pre-enzymatic hydrolysis treatment combined with further gastrointestinal digestion can enhance the functional value of plant proteins in the context of glycaemic control.

### 3.4. DPP-IV Inhibitory Activity

**Figure 4:**
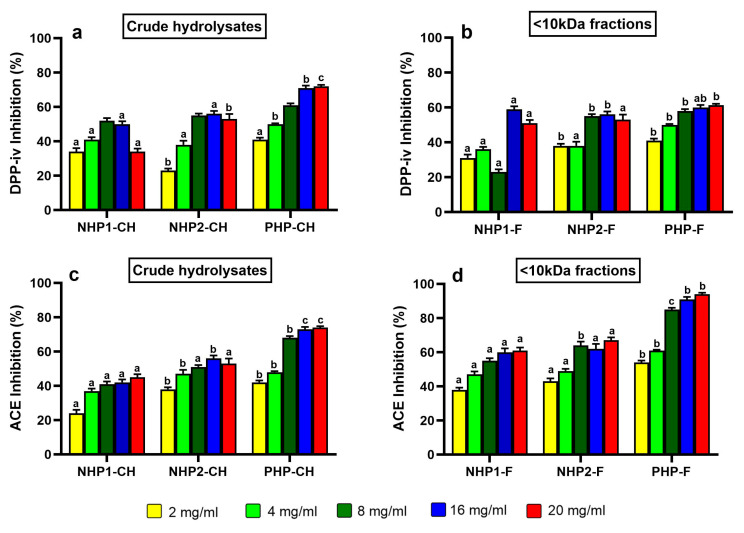
Inhibitory activity of crude hydrolysates NHP1-CH, NHP2-CH, and PHP-CH and <10 kDa peptide fractions of NHP1-F, NHP2-F, and PHP-F against DPP-IV (**a**,**b**) and ACE (**c**,**d**). Each bar represents the mean ± standard deviation (SD), (*n* = 3). Samples were tested at five concentrations. Different letters indicate significant differences (*p* < 0.05) between protein groups at the same concentration.

The dipeptidyl peptidase-IV (DPP-IV) inhibitory potential of crude and ultrafiltered (<10 kDa) hydrolysates was assessed across all pea protein samples. Among the tested hydrolysates, PHP-CH exhibited the most potent inhibition, reaching 71% at 5 mg/mL with an IC_50_ of 1.45 mg/mL (Figure 4a). This activity was notably higher than that observed in NHP1-CH and NHP2-CH, indicating that prior partial hydrolysis may enhance the release of peptides with DPP-IV inhibitory properties. For comparison purposes, the positive control Diprotin A exhibited over 90% inhibition at a concentration of 0.1 mg/mL, confirming assay reliability and highlighting the moderate but promising activity of pea-derived hydrolysates. Interestingly, ultrafiltration resulted in slightly reduced activity; the PHP-F fraction reached a maximum inhibition of 61% at 5 mg/mL with an IC_50_ of 2.49 mg/mL. In contrast, NHP1-F and NHP2-F showed marginal improvements over their crude forms (IC_50_ values of 2.29 mg/mL and 2.20 mg/mL, respectively), although without a strong dose-dependent trend (Figure 4b). This may be explained by the optimal bioactive peptide size being not very small, e.g., 3–10 kDa; pre-hydrolysis followed by ultrafiltration in PHP likely produced predominantly smaller peptides (<3 kDa), reducing their activity.

Whereas NHP1-F and NHP2-F may have retained or enriched mid-sized peptides (3–10 kDa), resulting in slight improvements in inhibition. This observation is consistent with findings by Han et al. (2021) [33], who reported that <3 kDa peptide fractions generated from sesame, soybean and flaxseed proteins did not exhibit higher DPP-IV inhibitory activity than the unfractionated hydrolysates. Nongonierma and FitzGerald [54] similarly emphasised the importance of evaluating both small and mid-size peptides (<1, 1–3, 3–10 and >10 kDa), as longer sequences can interact with enzyme binding pockets in unique ways, sometimes yielding synergistic effects.

### 3.5. ACE Inhibitory Activity

The angiotensin-converting enzyme (ACE) inhibitory activities of the pea protein hydrolysates and their ultrafiltered fractions were also evaluated. As shown in Figure 4c, a concentration-dependent inhibition was observed for all samples, with PHP again showing the most substantial effect. At 5 mg/mL, PHP exhibited 78% ACE inhibition with an IC_50_ of 3.1 mg/mL—significantly stronger than NHP1 and NHP2, which showed inhibition rates of 45% and 55%, respectively, and higher IC_50_ values (4.5 mg/mL and 4.1 mg/mL). Captopril, included as a positive control, exhibited >95% inhibition at 5 µg/mL, as expected given its purity and pharmacological potency.

Following ultrafiltration, PHP-F demonstrated enhanced ACE inhibitory activity, reaching 95% inhibition with an IC_50_ of 1.9 mg/mL (Figure 4d), indicating that bioactive peptides responsible for ACE inhibition are likely concentrated in the low molecular weight fraction. This supports the hypothesis that smaller peptides, particularly those containing hydrophobic or aromatic residues at the C-terminal position, are more efficient ACE inhibitors due to their compatibility with the enzyme’s zinc-binding active site [55,56].

These results are consistent with findings from other studies [13,57], where an increased ACE inhibition in legume-derived peptides following enzymatic hydrolysis and molecular weight fractionation was observed. In the current study, while DPP-IV inhibition appeared to benefit from the presence of a broader peptide range, ACE inhibition was clearly enhanced by the enrichment of smaller peptides.

### 3.6. Enhanced Bioactive Peptide Profiles and Predicted Enzyme Inhibition in PHP-F

The peptidomic profile of hydrolysed and non-hydrolysed pea protein samples revealed clear differences in peptide composition and predicted bioactivity across the four selected groups: NHP1-F, NHP2-F, PHP-CH, and PHP-F. Using LC-MS/MS combined with in silico analysis tools—BIOPEP-UWM and PeptideRanker—the ten most abundant peptides from each sample were analysed to predict their biological potential, as shown in Table 1. These tools provide insight into peptide functions based on structural motifs and bioactivity likelihood scores, thereby complementing the experimental enzyme inhibition assays [35].

**Figure 5:**
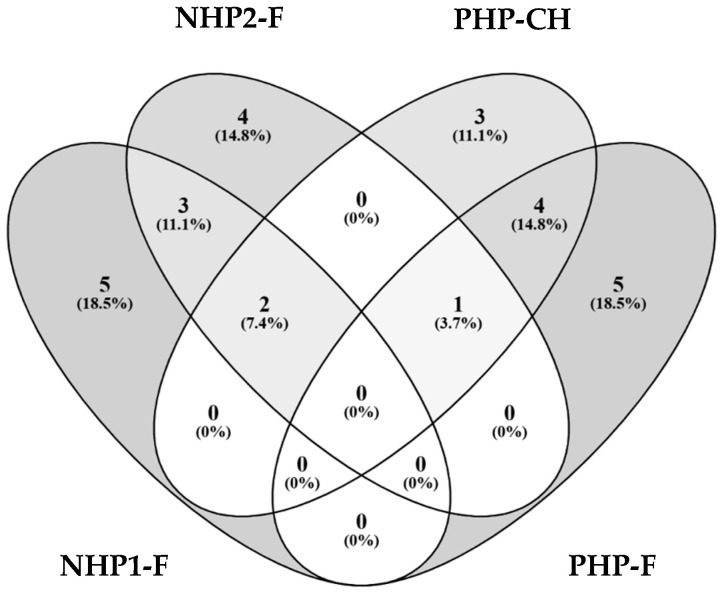
Venn diagram showing the distribution of the top 10 most abundant peptides identified in each sample (NHP1-F, NHP2-F, PHP-CH, PHP-F). Peptides were selected based on the highest intensity from LC-MS/MS data. The numbers represent the count and percentage of shared peptides between the different sample groups. Overlaps indicate common peptide sequences identified across multiple samples.

Among the non-hydrolysed fractions (NHP1-F and NHP2-F), five overlapping peptides were identified, including sequences such as FVPHYNL, RSRNPIYSNKFGKF, and RGIIPLEN (Figure 5). Several of these peptides have been previously reported to exhibit angiotensin-converting enzyme (ACE) inhibitory activity, suggesting a conserved pattern of antihypertensive potential in peptides derived from native pea storage proteins, such as vicilin and legumin [58,59].

The presence of similar sequences across untreated samples suggests that protein proteolysis may release bioactive fragments, although their inhibitory potential remained modest in vitro.

In contrast, PHP-CH and its ultrafiltered counterpart PHP-F presented unique profiles rich in medium-sized peptides with high predicted bioactivity. Several shared sequences—such as DQMPRRFY, LVIPVNGPGKF, and NIGPSSSPDIYNPEAGRIKTVTS—were predicted to exhibit strong inhibitory potential toward both α-amylase and α-glucosidase. These results align with previous studies indicating that controlled enzymatic hydrolysis can increase the yield of functional peptides targeting carbohydrate-digestive enzymes [13,60]. Moreover, the observation that these peptides were present in both the crude and ultrafiltered fractions supports the idea that functional sequences are enriched below the 10 kDa threshold, enhancing both their bioactivity and potential for intestinal absorption.

The PHP-F sample also yielded several unique peptide sequences—such as TETWNPNHPELK and WEAKGQTPLF—which, although previously unreported, were predicted using PeptideRanker to have high potential for enzyme inhibition, including DPP-IV and ACE. Notably, TETWNPNHPELK exhibited balanced physicochemical properties with a lower hydrophobicity index (33.3%) and higher solubility in aqueous media, while WEAKGQTPLF was more hydrophobic (50%), potentially affecting its solubility but not necessarily diminishing its bioactivity [61]. Additionally, some exclusive PHP peptides, like VQRYEARL and QRNALRRPYYSNAPQE, were associated with DPP-IV inhibition, reinforcing the in vitro results and corroborating earlier reports on legume-derived antidiabetic peptides [62,63].

Interestingly, although ultrafiltration is often used to enrich low molecular weight peptides with higher bioactivity, PHP-CH (crude hydrolysate) exhibited stronger DPP-IV inhibitory activity than its <10 kDa counterpart PHP-F. This may be explained by the presence of larger or mid-sized peptides present in PHP-CH that are partially excluded during filtration but contribute significantly to DPP-IV inhibition. For example, peptides such as FHMPPSSGSAPVNL and QRNALRRPYYSNAPQE, both present in PHP-CH, are rich in proline and aromatic residues—key features associated with DPP-IV inhibitory potential [64]. These longer peptides may contain internal dipeptide motifs (e.g., Ile-Pro, Val-Pro) or adopt conformations favourable for enzyme binding. The presence of serine-rich segments and multiple potential interaction sites may facilitate multi-point binding to the DPP-IV active site, enhancing inhibitory potency. Its absence in the ultrafiltered PHP-F fraction suggests that it was removed during the <10 kDa filtration step, potentially explaining the slightly reduced DPP-IV inhibition observed in PHP-F compared to PHP-CH [65].

In silico analysis revealed stronger predicted inhibition scores for ultrafiltered samples—particularly PHP-F—compared to their crude counterparts. This trend was consistent with the in vitro results, where PHP-F showed enhanced inhibitory activity against ACE and α-amylase. These predictions likely reflect the presence of multiple bioactive peptides within each fraction, suggesting that observed effects may result from the synergistic action of several peptides rather than isolated sequences alone [13].

Overall, the combined LC-MS/MS and in silico profiling demonstrated that enzymatic hydrolysis and membrane ultrafiltration significantly influence the release, enrichment, and identification of bioactive peptides. The approach confirmed the release of specific peptide sequences capable of inhibiting enzymes relevant to glycaemic regulation (α-amylase, α-glucosidase, DPP-IV) and hypertension (ACE), supporting the potential of hydrolysed pea protein as a functional food ingredient. The results also align with prior literature emphasising the value of bioinformatic tools as pre-screening steps for the development of protein hydrolysates with enhanced traits [22,66]. However, given the complexity of peptide mixtures, future work is needed to validate the predicted bioactivities of individual peptides experimentally, as synergistic or antagonistic effects within the peptide pool may influence their true functional potential. Additionally, further characterisation of their structural properties, molecular docking and dynamics studies are necessary to elucidate the mechanisms between peptides and target proteins. All peptides identified across the four samples with a high predicted bioactivity score (>0.5) are provided in Supplementary Table S1, structural features of the most 10 abundance peptides are summarised in Supplementary Table S2 and predicted bioactivities detailed in Supplementary Table S3.

## 4. Conclusions

Pre-hydrolysis significantly improved the susceptibility of pea protein to pepsin digestion, resulting in enhanced solubility, peptide release, and enzyme-inhibitory properties. The pre-hydrolysed pea protein sample showed greater potential to inhibit α-amylase, α-glucosidase, and dipeptidyl peptidase-IV enzymes, likely due to the enrichment of medium-sized molecular weight peptides with bioactive potential. In contrast, the non-hydrolysed proteins were less efficiently digested and exhibited weaker inhibitory activity. These findings demonstrate the benefits and advantages of controlled enzymatic processing in enhancing the functionality of plant proteins for their potential use in metabolic health management. For future studies, it is recommended to chemically synthesise the identified peptides and validate their bioactivity and mechanisms of action by means of in vitro and in vivo assays.

## Figures and Tables

**Table 1:** Identified peptides from pea protein samples and their predicted enzyme inhibitory activities based on in silico analysis by means of BIOPEP.

Protein Sample	Peptide Sequence	ACE Inhibition	DPP-IV Inhibition	α-Amylase Inhibition	α-Glucosidase Inhibition
**NHP1-F**	FVPHYNL, RSRNPIYSNKFGKF, RGIIPLEN, RSDQENPFIF, LAIPVNKPGQL, YKSKPHTIF, YRNGIYAPHW, YENENGHIRL, IRRTIDPNGLHL, FENENGHIRL	All	All	None	None
**NHP2-F**	NENGHIRL, RSRNPIYSNKFGKF, RGIIPLEN, FVPHYNL, LAIPVNRPGQL, YKSKPHTIF, LVIPVNGPGKF, LAIPVNKPGQL, KVLYGPTPVRDGF, YRAKPHTIF	All	All	None	None
**PHP-CH**	FVPHYNL, RSRNPIYSNKFGKF, LVIPVNGPGKF, FHMPPSSGSAPVNL, DQMPRRFY, VQRYEARL, EITPEKNQQL, QRNALRRPYYSNAPQE, SHKPEYSNKFGKL, NIGPSSSPDIYNPEAGRIKTVTS	All	All	DQMPRRFY	FHMPPSSGSAPVNL, NIGPSSSPDIYNPEAGRIKTVTS, DQMPRRFY, VQRYEARL, EITPEKNQQL, QRNALRRPYYSNAPQE, SHKPEYSNKFGKL
**PHP-F**	LVIPVNGPGKF, AIPVNKPGQLQ, DQMPRRFY, FHMPPSSGSAPVNL, NIGPSSSPDIYNPEAGRIKTVTS, SHKPEYSNKFGKL, TETWNPNHPELK, WEAKGQTPLF, PRRFYRP, KPEYPYSN	All	All	DQMPRRFY PRRFYRP	AIPVNKPGQLQ, DQMPRRFY, FHMPPSSGSAPVNL, NIGPSSSPDIYNPEAGRIKTVTS, SHKPEYSNKFGKL, TETWNPNHPELK, WEAKGQTPLF, KPEYPYSN, PRRFYRP

## Data Availability

The original contributions presented in this study are included in the article/Supplementary Materials. Further inquiries can be directed to the corresponding author.

## Supplementary Materials

The following supporting information can be downloaded at https://www.mdpi.com/article/10.3390/foods14193306/s1, Table S1: Peptides identified across the four samples (NHP1-F, NHP2-F, PHP-CH, PHP-F) with predicted bioactivity scores > 0.5; Table S2: Structural features of the 10 most abundant peptides (sequence, molecular weight, charge, hydrophobicity); Table S3. Predicted bioactivities of identified peptides based on in silico analysis.

## Abbreviations

The following abbreviations are used in this manuscript:

DPP-IV	Dipeptidyl peptidase
ACE	Angiotensin converting enzyme
DH	Degree of hydrolysis
PHP	Pre-hydrolysed pea protein
NHP	Non-hydrolysed pea protein
